# Aberrations of DNA Repair Pathways in Prostate Cancer—The State of the Art

**DOI:** 10.3390/ijms24054301

**Published:** 2023-02-21

**Authors:** Stergios Boussios, Matin Sheriff

**Affiliations:** 1 Department of Medical Oncology, Medway NHS Foundation Trust, Windmill Road, Gillingham ME7 5NY, UK; 2 Faculty of Life Sciences & Medicine, School of Cancer & Pharmaceutical Sciences, King’s College London, London SE1 9RT, UK; 3 Kent Medway Medical School, University of Kent, Canterbury CT2 7LX, UK; 4 AELIA Organization, 57001 Thessaloniki, Greece; 5 Department of Urology, Medway NHS Foundation Trust, Windmill Road, Gillingham ME7 5NY, UK

Prostate cancer (PC) is the second most commonly diagnosed cancer in males worldwide and the fifth most common cause of cancer-related death in men [1]. The incidence of metastatic PC has increased as has the incidence of localized PC, which is correlated with a variety of genetic, hereditary and environmental factors, including older age, family history of PC and African ethnicity. This Special Issue focused on—but was not only limited to—the state of the art in the aberrations of DNA damage repair (DDR) pathways in PC, given that large-scale sequencing efforts have resulted in a better understanding of the genomic landscape of PC.

Synthetic lethality represents the therapeutic strategy of the “BRCAness” molecular signature, demonstrating the shared phenotype between sporadic and familial cancers with *BRCA1* and *BRCA2* mutations [2]. This is based on the evidence that tumors with a deficiency in additional genes implicated in homologous recombination may also respond to treatment in a similar manner as *BRCA1*- and *BRCA2*-mutated tumors. Proteins involved in homologous recombination repair include CDK12, ATM, FANCD2, RAD51C, CHEK2, PALB2, BRIP1 and HDAC2. Consequently, alterations in DDR genes, particularly in those involved in homologous recombination repair, are predictors of a response to poly (ADP-ribose) polymerase (PARP) inhibition. Whilst carriers of germline mutations in *BRCA1* and *BRCA2* genes are known to have a lifetime risk of ovarian cancer of 35–60% and 12–25%, respectively, it has more recently been established that germline or somatic aberrations in the DDR genes are present in 19% of cases of primary PC and in approximately 23% of metastatic castration-resistant PC cases [3,4]. The incidence of germline *BRCA1* and *BRCA2* gene mutations in newly diagnosed PC is 1.2–2%. *BRCA1* and *BRCA2* gene carriers can lead to an increase in the risk of developing PC that is around 4- and 8-fold, respectively [5]. Within this context—apart from breast and ovarian cancer—several PARP inhibitors have been investigated in metastatic castration-resistant PC patients [6,7,8]. The PARP inhibitors currently approved by the US Food and Drug Administration (FDA) for the treatment of metastatic castration-resistant PC are olaparib, rucaparib and niraparib [9]. Differences exist in their metabolism; olaparib and rucaparib are metabolized by cytochrome P450 enzymes, whilst niraparib is metabolized by carboxylesterase-catalyzed amide hydrolysis. The synthetic lethality mechanism of action may have a protective effect against severe PARP inhibitor toxicity [10].

The treatment of PC has rapidly changed. Namely, androgen receptor (AR) signaling inhibitors downregulate DDR gene expression and increase DNA damage, maximizing the efficacy of PC to PARP inhibitors. Androgen deprivation therapy (ADT) formulates a status of “BRCAness” when PARP and AR signaling are concurrently inhibited. As such, PARP inhibitors may be effective even beyond DDR mutated PC [11]. Furthermore, given that micro-vessel density represents a predictor of metastasis, targeting angiogenesis is an area of ongoing research [12].

The majority of PC cases are diagnosed and treated with localized disease; nevertheless, some patients have metastatic PC, either at presentation or following localized disease [13]. From a therapeutic point of view, those with high-risk non-metastatic PC receive ADT for 3 years, which may be combined with radiotherapy. Recently, it has been reported that abiraterone alone or in combination with enzalutamide with ADT led to significantly higher rates of metastasis-free survival versus ADT alone [14]. There is robust evidence indicating that AR activates the DDR pathways, which provides a rationale for the use of ADT with stereotactic ablative radiotherapy for hormone-sensitive oligometastatic PC [15].

Biomarkers play an important role in the selection of patients that may benefit from a particular type of treatment. Within this context, microRNAs, AR variants, bone metabolism, and neuroendocrine and metabolite biomarkers are promising candidates, which are crucial to identify in the era of the precision medicine [16]. Teng PC et al. demonstrated a rigorous bioinformatic pipeline using publicly available platforms and databases to develop a DDR-based panel that could aid with PC prognostication [17]. The study suggested that a four-gene panel consisting of *EXO1*, *DNTT*, *NEIL3* and *EME2* is a promising DDR-derived biomarker that should be further investigated. Depending on the cellular context, autophagy could play either a detrimental or a protective role in PC survival. There is ongoing research on autophagy modulation in vitro and on whether it may potentially serve as a biomarker. This can open up new therapeutic avenues in PC therapy and may also optimize the prognostic stratification of patients [18]. The somatostatin, cortistatin and somatostatin receptor system is an additional source of prognostic biomarkers and therapeutic targets for the subset of endocrine-related cancers [19]. This system inhibits multiple processes including hormone secretion, as well as cell proliferation, migration and invasion. Sáez-Martínez P et al. reported that the treatment with somatostatin and cortistatin peptides may reduce proliferation, migration and colony formation only in androgen-independent PC cells (22Rv1 and PC-3 cells), but not in normal prostate and androgen-dependent PC cells. This supports the idea of the potential and specific antitumor capacity of these peptides in the most aggressive castration-resistant PC [20]. Proteomic technologies, such as mass spectrometry and protein array analysis, have advanced the dissection of the underlying molecular signaling events and the proteomic characterization of several cancers, including PC [21,22]. Zhong et al. compiled a comprehensive imaging resource to complement high-throughput proteomic and genomic data [23]. Therefore, there now exists a large number of shared data resources with excellent potential for reuse in both biomedical and computational studies.

N-Myc is not significantly expressed in adult tissues; nevertheless, it was reported to be amplified and overexpressed in neuroendocrine PC tumors. The alteration and deregulation of N-Myc induced tumor proliferation and progression, resulting in a low survival rate in neuroendocrine PC patients [24]. The Myc protein represents a “slippery as an eel” target, and this is the reason for the indirect pharmacological approaches that target its transcription, translation or degradation [25]. Ton AT et al. identified compound VPC-70619, which blocks the N-Myc-Max heterocomplex from binding to DNA E-boxes and demonstrated strong inhibition activity against N-Myc-dependent cell lines, along with high bioavailability in both oral and intraperitoneal routes [26].
